# Molecular recognition of *Escherichia coli* R1-type core lipooligosaccharide by DC-SIGN

**DOI:** 10.1016/j.isci.2024.108792

**Published:** 2024-01-04

**Authors:** Ferran Nieto-Fabregat, Angela Marseglia, Michel Thépaut, Jean-Philippe Kleman, Massilia Abbas, Aline Le Roy, Christine Ebel, Meriem Maalej, Jean-Pierre Simorre, Cedric Laguri, Antonio Molinaro, Alba Silipo, Franck Fieschi, Roberta Marchetti

**Affiliations:** 1 Department of Chemical Science, University of Naples Federico II Via Cinthia 4, 80126 Naples, Italy; 2 University Grenoble Alpes, CNRS, CEA, Institut de Biologie Structurale, 41 Avenue des Martyrs, 38000 Grenoble, France; 3 Institut Universitaire de France (IUF), Paris, France

**Keywords:** Microbiology, Structural biology

## Abstract

Due to their ability to recognize carbohydrate structures, lectins emerged as potential receptors for bacterial lipopolysaccharides (LPS). Despite growing interest in investigating the association between host receptor lectins and exogenous glycan ligands, the molecular mechanisms underlying bacterial recognition by human lectins are still not fully understood. We contributed to fill this gap by unveiling the molecular basis of the interaction between the lipooligosaccharide of *Escherichia coli* and the dendritic cell-specific intracellular adhesion molecules (ICAM)-3 grabbing non-integrin (DC-SIGN). Specifically, a combination of different techniques, including fluorescence microscopy, surface plasmon resonance, NMR spectroscopy, and computational studies, demonstrated that DC-SIGN binds to the purified deacylated R1 lipooligosaccharide mainly through the recognition of its outer core pentasaccharide, which acts as a crosslinker between two different tetrameric units of DC-SIGN. Our results contribute to a better understanding of DC-SIGN-LPS interaction and may support the development of pharmacological and immunostimulatory strategies for bacterial infections, prevention, and therapy.

## Highlights

• DC-SIGN is able to recognize *E. coli* surface presenting R1 type core oligosaccharides
• The binding occurs through the recognition of the LOS outer core pentasaccharide
• We suggested that OSR1 can act as a crosslinker between 2 different DC-SIGN tetramers
• Our outcomes contribute to a better understanding of bacterial recognition by lectins

## Graphical abstract

**Figure:**
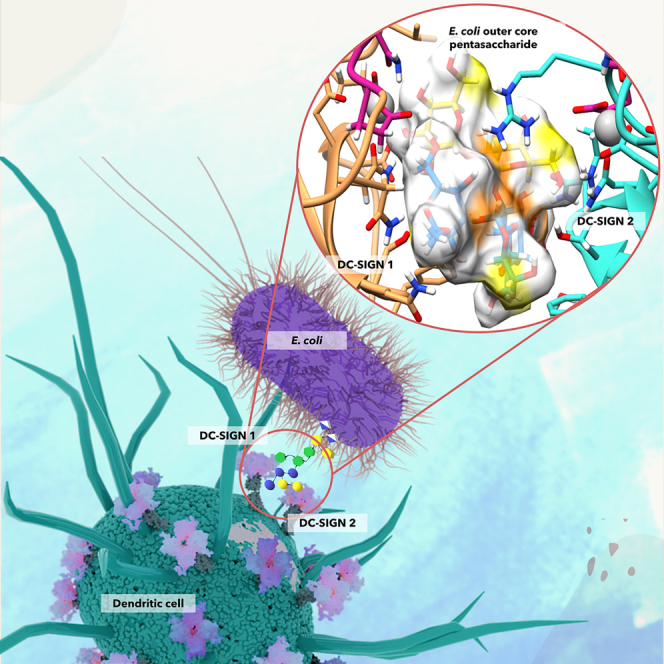

## Introduction

Lipopolysaccharides (LPS) are peculiar glycolipids which represent the major components of the external leaflet of the gram-negative bacteria outer membrane.^1^^,^^2^ They are heat stable amphiphilic molecules, which consist of three structurally and genetically distinct domains: the lipid A, integrated in the outer membrane; the core oligosaccharide (OS), in turn composed of inner and outer core regions; and the distal O-specific polysaccharide (O-PS) chain, that extends outwards the bacterial surface. In the smooth form of the LPS, the core OS links the highly conserved lipid A portion to the hypervariable antigenic O-polysaccharide; on the contrary, rough LPSs are devoid of the O-antigen. Notably, several pathogenic gram-negative bacteria expose on their surface, as main glycolipids, lipooligosaccharides (LOSs) lacking the O-antigen.

Research in the past decades has clearly showed that the LPSs are the most potent microbial product able to boost the innate immunity in eukaryotes and in humans able to drive to cytokine storm and death by sepsis. Due to their capacity of triggering the host immune system, they are considered potent pathogen-associated molecular patterns (PAMPs), playing a key role in the pathogenesis of gram-negative infections.^3^^,^^4^^,^^5^ In particular, the lipid A portion is well known to be the main immunostimulatory center of LPS, able to modulate the immune response upon its recognition by the MD-2/TLR4 receptorial complex. More recently, other pattern recognition receptors (PRRs), including transient receptor potential (TRP) channels and caspases, have been identified as sensors of extracellular and intracellular LPS.^6^^,^^7^

While enormous efforts have been devoted to the elucidation of lipid A recognition by host immune system, shedding light on the correlation between lipid A structure and activity,^6^ less is known about the interaction and immune recognition of LPS saccharidic regions (O-antigen and core OS) by host PRR (PRRs). Actually, intense research in the field of innate immunity has unveiled the ability of the host immune system to respond to gram-negative bacteria thanks to carbohydrate sensing macromolecules, *inter alia*.^8^ However, both molecular insights and effects of these PRR-LPS interactions is far to complete. Moreover, it is easy to assume that there are unknown or uncharacterized receptor(s) with the ability to recognize O-PS and/or OS structures in bacterial LPS. Direct experimental data are thus strongly needed to advance our understanding of the molecular mechanisms laying at the basis of bacterial LPS recognition by the host immune system, contributing to the development of novel pharmacological and immunostimulatory strategies for the prevention and therapy of bacterial infections.

In innate immunity, lectins often act as PRRs. They constitute a broad group of non-immunoglobulin proteins, occurring ubiquitously in nature, with high affinity for carbohydrates, without displaying any enzymatic activity.^9^^,^^10^ Lectins play important roles in the innate immune system being involved in cell-cell communication, cellular trafficking, and regulation of the immune cell functions,^11^ thus, making them potential therapeutic agents. Moreover, due to their ability to recognize carbohydrate structures, they have emerged as potential receptors for the LPS carbohydrate moieties.^8^^,^^12^

The family of C-type lectins (CTL) is currently the biggest and most diverse class of human lectins. It is composed of transmembrane and soluble receptors with the ability of recognizing specific glycan structures, through their carbohydrate-recognition domain (CRD),^13^ usually in a Ca^2+^ dependent manner. One of the main representatives of transmembrane CTLs is the dendritic cell-specific intracellular adhesion molecules (ICAM)-3 grabbing non-integrin (DC-SIGN) also known as CD209 (cluster of Differentiation 209).^14^ This protein is found on macrophages, monocytes, and is mainly expressed by dendritic cells (DC) which act as potent phagocytic cells, because of DC-SIGN, mediating the adherence and phagocytosis of different bacterial strains^15^^,^^16^ thus playing a crucial role in defending the host against invading pathogens. However, interactions between DC-SIGN and bacterial glycans do not always assist host defense against detrimental microorganisms; feared pathogens, as *Mycobacterium tuberculosis*,^17^ can indeed exploit the interaction with DC-SIGN to subvert some DC roles.

DC-SIGN belongs to the mannose receptor family and is expressed as a tetramer on the cell surface, with each monomer composed by a single CRD, a neck region and an intracellular domain.^15^ The extended neck in the extra-cellular domain is pivotal in the oligomerization of the CRD further influencing the binding properties of the protein. It is characterized by an EPN (Glu-Pro-Asn) motif that allows the Ca^2+^ dependent binding to bacterial glycans through structures including fucose (Le^a^, Le^b^, Le^X^, Le^Y^, and sulfo- Le^a^) and mannose residues. It also serves as a receptor of the human immunodeficiency virus type 1 (HIV-1) gp120. Structural studies also showed the ability of the protein to strongly bind to GlcNAc containing structures.^18^^,^^19^^,^^20^

As a result of being able to discriminate among multiple ligands, some studies highlighted the peculiarity of DC-SIGN in modulating the signaling of TLRs into a pro-initiation of the immune response, upon recognition of mannose-containing structures, or in an anti-inflammatory response upon the recognition of fucosylated glycans, maintaining the immunological homeostasis.^15^^,^^21^^,^^22^^,^^23^^,^^24^^,^^25^

The role of DC-SIGN in the phagocytosis of several gram-negative bacteria including *E. coli*^26^ has been previously reported.^18^^,^^25^^,^^27^^,^^28^ In detail, it has been showed that the DC-SIGN induced phagocytosis of *E. coli* occurs in the absence of O-antigen polysaccharides, and in the presence of a complete core OS,^19^ however, the molecular features driving these interactions are still missing. Thus, with the aim to extend our knowledge of microbial glycans recognition by the host immune system, in this study, we dissected the binding between DC-SIGN and the LOS exposed on the surface of *E. coli*. Precisely, among the five *E. coli* core structures (termed K-12 and R1-R4),^29^ we focused our attention in investigating the interaction of DC-SIGN with the most prevalent in clinical isolates, the core OS R1. In particular, the deacylated LOS from R1, constituted by the R1 type core OS and the lipid A sugar backbone, namely OSR1, was purified and studied in the interaction with DC-SIGN by using fluorescence microscopy, surface plasmon resonance (SPR), spectroscopic, and computational techniques.^30^^,^^31^ This integrated approach allowed us to define the region of the core OS recognized by and bound to DC-SIGN and propose a 3D model for the interaction.

## Results

### Fluorescence microscopy

**Figure 1:**
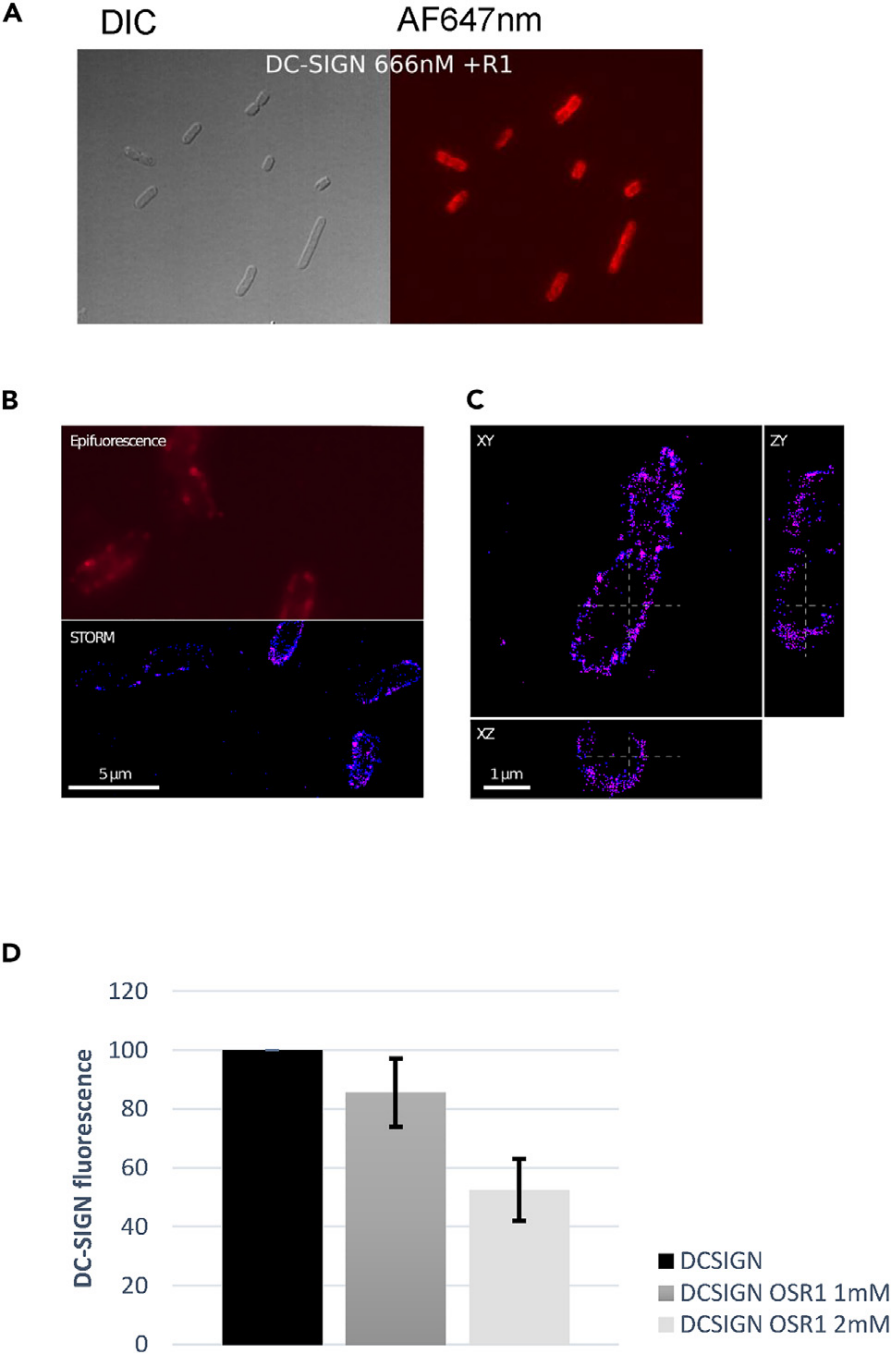
DC-SIGN ECD binds strongly to R1 core oligosaccharide at the surface of *E. coli* (A) Differential Interference Contrast (DIC) and epifluorescence imaging of R1-cells incubated with DC-SIGN ECD and extensively washed before imaging. STORM imaging of DC-SIGN ECD labeled with AF647 on *E. coli* R1 cells. (B) Split panel showing wide field epifluorescence and STORM reconstructed imaging of the labeled bacteria. (C) Orthogonal plane projections (according to the dashed lines) of a single bacterium from the field. A cylindrical lens is used to provide the 3D localization precision. (D) Flow cytometry quantification of DC-SIGN ECD labeled with AF647 bound to R1-cells in absence or presence of 1 and 2 mM OSR1. 50% binding inhibition is achieved with 2 mM OSR1. Experiments were done in triplicates and standard deviation is shown.

The association between *E. coli* cells and DC-SIGN was initially assessed by epifluorescence microscopy. *E. coli* F470 strain was chosen as the typical strain carrying the R1 type OS, abundant in clinically relevant *E. coli* and carrying no O-antigen. DC-SIGN was labeled with Alexa Fluor 647, incubated with *E. coli* strain F470 cells, namely R1-cells, and excess protein was washed extensively. A strong fluorescence is observed on the bacteria that account for the binding of DC-SIGN onto R1-cells (Figure 1A). In order to visualize more precisely DC-SIGN binding on *E. coli*, STochastic Optical Reconstruction Microscopy (STORM) was applied to DC-SIGN/R1-cells interaction. This microscopy method abrogates the light diffusion resolution limit of microscopy thanks to the blinking characteristics of the fluorophore that is maintained during the experiment by depleting free oxygen in the buffer. Acquisition of serial images of bacteria labeled with DC-SIGN-AF647 over minutes allow to visualize individual fluorophores fluorescence emission (Figure 1B). STORM imaging of DC-SIGN labeled with AF647 on R1-cells clearly showed that the C-type lectin was able to strongly bind to R1-cells core OS at the surface of *E. coli.* The specificity of the binding has been assessed by flow cytometry experiments in the presence of increasing concentration of OSR1 (Figures 1D and S1).

### LOS core oligosaccharide isolation and DC-SIGN recombinant expression

**Figure 2:**
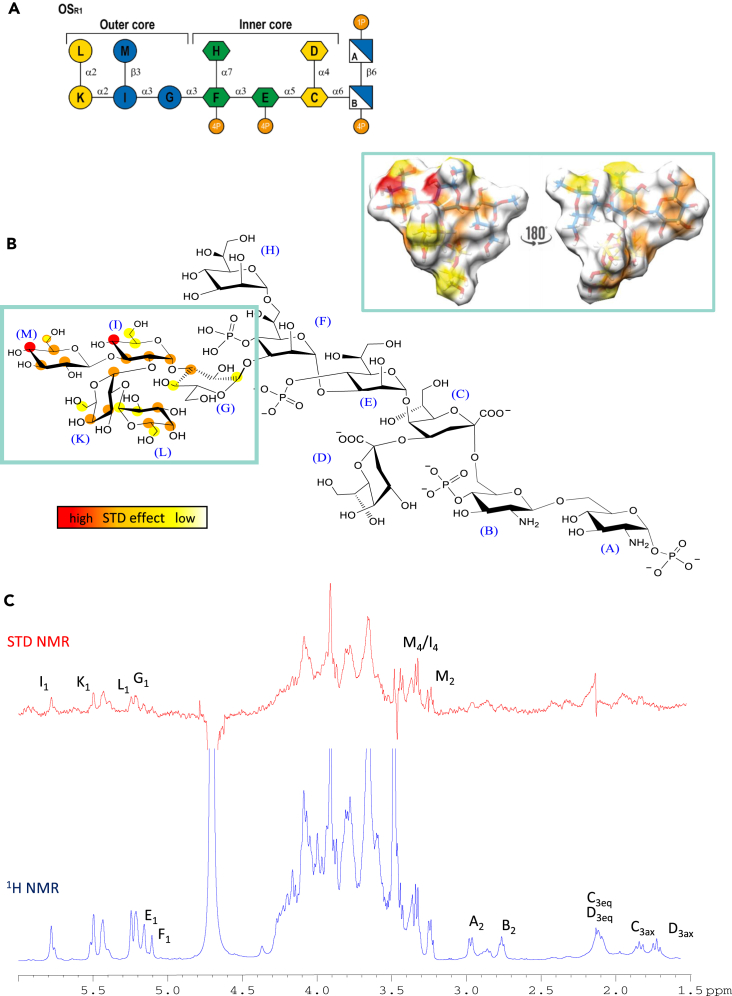
Saturation transfer difference NMR studies show the recognition of OSR1 by DC-SIGN through the outer core pentasaccharide (A) OSR1 schematic structure using symbol-nomenclature for glycans (SNFG). (B) STD-derived epitope mapping of the interacting oligosaccharide with color coding from the highest (red) to the lowest (yellow) observed STD effect and the 3D representation of the STD-derived epitope mapping. (C) ^1^H NMR reference spectrum at the bottom (blue) with the 1D STD NMR spectrum on top (red) of the 1:90 mixture for DC-SIGN: OSR1 complex with some of the key proton resonance signals labeled.

In order to accurately examine the interaction between LOS from R1-cells and DC-SIGN, we thus isolated the two partners of the interaction. In detail, we extracted and purified the lipooligosaccharide from bacterial cells and isolated its deacylated form containing the core OS and the lipid A sugar backbone (Figure 2A). It is a dodecasaccharide composed of two residues of galactose and three glucose units in the outer core region and three L-*glycero*-D-manno-heptoses (two of them phosphorylated at position 4) and two 3-deoxy-D-manno-oct-2-ulosonic acids (Kdo), in the inner core portion; the two glucosamine residues (phosphorylated one at position 1 and the other at position 4) at reducing end belong to the lipid A moiety.^4^ Moreover, we expressed and purified the extracellular domain of DC-SIGN, namely DC-SIGN extra cellular domain (ECD) and its CRD, namely DC-SIGN CRD. We then used SPR, NMR spectroscopy, and computational techniques (docking and molecular dynamics) to explore the molecular basis of the interaction between DC-SIGN and OSR1 building and validating a model of the 3D ligand-protein complex.

### SPR analysis

**Figure 3:**
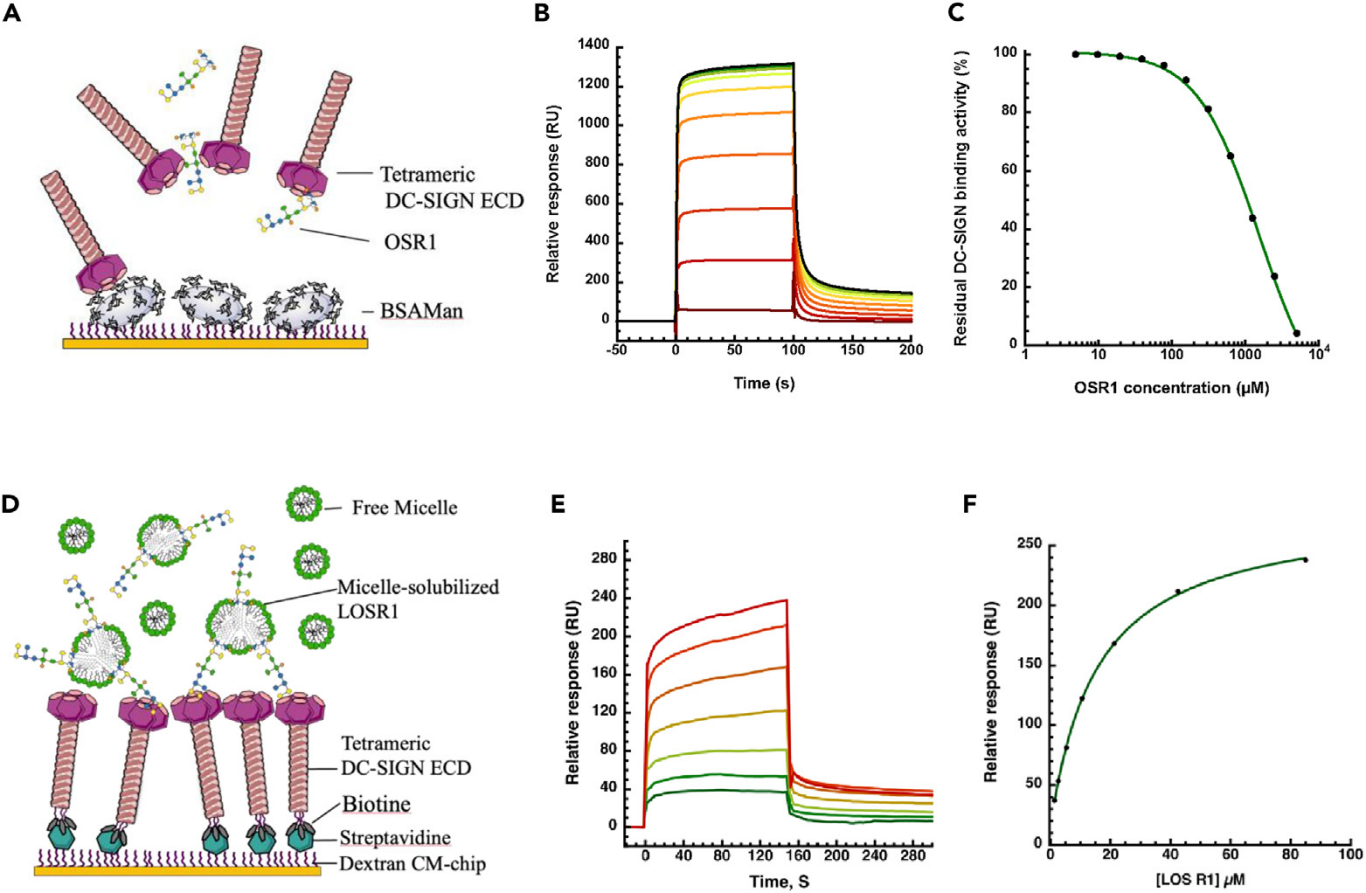
Analysis of DC-SIGN interaction with OSR1 by surface plasmon resonance (A) Principle of competition experiment. (B) DC-SIGN ECD (20 μM) was incubated without or with OSR1 at increasing concentrations from 5 μM to 5 mM (from dark green to dark red)). The samples were co-injected over BSA-Mannotriose surface. (C) The steady state responses were extracted from the sensorgrams (in B), converted to DC-SIGN ECD residual activity, and plotted against OSR1 concentration. The inhibition curves were then fitted using four-parameter logistic model. The experiment was done twice using two distinct surfaces. (D) Principle of direct interaction of R1-cells LOS/DDM Micelle with DC-SIGN ECD oriented surface. (E) R1-cells LOS incorporated in DDM micelle have been injected onto DC-SIGN ECD at increased concentrations from 2.6 to 85 μM (serial dilution within running buffer by a factor of 2 from dark red to dark green). (F) The steady state binding responses from the sensorgram (in E) were plotted against LOSR1 concentration.

The ability of DC-SIGN ECD to recognize OSR1 was evaluated by SPR analysis. In detail, the affinity of the OSR1 for DC-SIGN has been estimated thanks to the use of a classical competition assay. Tetrameric extracellular domain of DC-SIGN was injected over a sensorship surface functionalized with a mannosylated BSA glycoconjugate. This interaction has been challenged with increasing concentration of OSR1 leading to inhibition of DC-SIGN interaction with the surface on a concentration dependence manner (Figure 3B). The inhibitory curve resulting from this competition experiment (Figure 3C) allowed to evaluate a mean IC_50_ of 1.059 ± 0.003 mM. Thus, SPR experiments demonstrated the interaction between OSR1 and DC-SIGN. The affinity observed was significantly higher than those would have been expected from terminal galactose or glucose, as present here in position **L** and **M** (Figure 2A), that are not very strong ligands of DC-SIGN. As a reference, using such a competition test, mannose monosaccharide or OS with terminal mannose classically provide an IC_50_ around 3 mM. Glucose and galactose would provide IC_50_ just below 10mM or even 30 mM, respectively.^32^ Thus, the IC_50_ of 1 mM observed strongly suggest a larger epitope of interaction, as further confirmed by the NMR analysis. In this SPR competition test, DC-SIGN ECD was free in solution, not embedded in a cell membrane and exposed at the cell surface as it is in a physiological situation. Thus, DC-SIGN ECD has much less constraint. It is also the case for OSR1 extracted from the LPS and now as a soluble diffusible ligand.

To better mimic the physiological conditions of such interaction, we have recently developed a direct interaction test, using SPR, where C-type lectin receptor is functionalized in an oriented way.^33^ Moreover, we solubilized purified R1 LOS in detergent allowing their solubilization and presentation inserted within a mixed micelle (LPS/DDM). Such R1 LOS containing micelles have been used for direct interaction study and injected onto DC-SIGN oriented surface (Figure 3E) at different concentration of LOS. From the sensorgrams obtained a titration curve has been traced (Figure 3F) and allowed to evaluate a K_Dapp_ = 15,6 μM. From the OSR1 in competition test to the R1 LOS tested in direct interaction on an oriented surface, we observed an increase of affinity by several orders of magnitude (from IC_50_ of 1mM to K_Dapp_ of 15 μM). This suggested that statistically micelles containing several LOS can generate avidity through multivalency on the DC-SIGN oriented surface. In addition, we cannot exclude the possibility that the lipid A moiety of the LOS might contribute also to this improved binding strength.

### NMR analysis

To deeply explore the molecular interaction between DC-SIGN ECD and OSR1, the binding was also evaluated by saturation transfer difference NMR (STD NMR).^10^^,^^34^ The STD NMR results supported the ability of DC-SIGN ECD to recognize the OSR1 as indicated by the several enhancements observed in the STD NMR spectrum acquired upon the addition in solution of the recombinant form of the protein (the same used for the SPR experiments). The analysis of the less crowded regions of the spectrum, primarily the one containing the anomeric signals, and the comparison of both multiplicity and intensity of the STD NMR signals with respect to the reference spectrum, revealed an extended binding epitope involving the outer core region, in accordance with the binding affinity derived by the SPR analysis (Figure 2B). Specifically, the anomeric protons of **I**, **K**, **L,** and **G** sugars (Figure 2C) gave rise to remarkable STD effects; on the contrary, the resonances of **E** and **F** residues were not observed in the STD NMR spectrum, thus suggesting the outer core moiety as the one mainly involved in the binding with DC-SIGN. Accordingly, the most intense signals corresponded to the H4 of glucose residues **M** and **I.** Also protons H2, H3, and H5 of **M** strongly contributed to the interaction. An intermediate contribution was observed for protons H1, H2, and H3 of glucose **I** and H3, H4, and H5 of galactose **L**. Lower STD enhancements were observed for signals belonging to some protons of the galactose residues **K** and **L** and glucose **G**. For **K**, H1, H3, and H4 exhibited a higher contribution than H6. On the other hand, H3 of **G** showed a higher contribution than H1 and H4 of the same residue. The other protons of these two saccharides (**K** and **G**) did not seem to have an interaction with the protein. Moreover, signals belonging to the inner core residues were not observed as indicated for example by the absence of STD NMR signals either of the diastereotopic methylene protons of Kdo residues (**C** and **D**) and of the protons at position 2 of the glucosamine residues **A** and **B** of the lipid A, further suggesting the main contribution of the outer core to the interaction of OSR1 with DC-SIGN.

With the aim to localize the binding site of LOS and to get more details into the protein residues involved in the recognition process, we decided to carry out protein-based NMR experiments. ^15^N labeled DC-SIGN CRD was produced and its binding to OSR1 has been analyzed by ^1^H-^15^N NMR spectroscopy. Unfortunately, 2D ^1^H-^15^N correlation experiments did not show significant chemical shift perturbations on DC-SIGN CRD upon the addition of 20 M equivalents of the OS in solution (Figure S2). This suggests a very low affinity of the protein CRD for the core OS, likely due to the different conformation/arrangement in solution of the recombinant CRD with respect to the ECD.

### Molecular modeling analysis

Based on such premises and given the presence of isochronous NMR signals, which hindered a quantitative assignment of all the STD NMR effects, hampering the accurate definition of the ligand epitope mapping, computational studies were used to endorse the obtained experimental results and depict a 3D model of protein-ligand complex. By applying a reductionist approach the pentasaccharide formed by the five outer core residues (**G**, **I**, **K**, **L,** and **M**) from OSR1, built with the GLYCAM^35^ carbohydrate builder, was used as ligand to study its interaction with a single monomeric DC-SIGN subunit, extracted from PDB 1K9I. Docking calculations by means of Autodock 4.2^36^ thus provided a first prediction of the protein-ligand interactions in the modeled complex, allowing to select representative poses from the most populated clusters as starting point for a molecular dynamic (MD) simulations in explicit solvent with AMBER.

Given that the whole outer core seemed to be recognized by means of NMR, we firstly assumed that the protein should have a binding pocket big enough to accommodate the full pentasaccharide. However, according to the results of the computational studies, the five sugars of the outer core could not simultaneously interact with the protein binding pocket as half of them appeared to be solvent exposed. Considering that STD NMR experiments can be sensitive to different ligand orientations in the protein binding pocket,^37^^,^^38^ we postulated a second hypothesis based on the possibility of having multiple binding modes. Actually, the docking results already presented two interesting poses for OSR1 pentasaccharide recognition by DC-SIGN ECD. The less energetic cluster (cluster A) displayed residues **L** and **G** in the protein binding site (Figure S3) while the most populated cluster (cluster B) presents **K**, **I**, and **M** accommodated inside the pocket (Figure S4). The best representative poses from the different orientations of the ligand (taking always into account that the chain extends through residue **G**) were used for running MD simulations. Regarding cluster A, although the **L** residue could be found coordinating with the Ca^2+^ ion, after a few nanoseconds the ligand left the binding pocket, resulting in a non-stable MD (Figure S3B). For the cluster B, something less expected happened; after a few nanoseconds the ligand shifted toward a polar region instead of interacting with the expected residues in the protein binding pocket, establishing contacts with Asn362, Arg345, and Asn344 trough residue **K**, with Ser360 through unit **I**, and with Asn362 and Asn311 via **M** (Figure S5). Thus, the obtained results indicated that the ligand was not stable in the principal calcium-dependent binding pocket, suggesting that the different binding modes hypothesis was not feasible.

Structurally, DC-SIGN CRD is constituted by two α-helices and five β-strands. The loop that extends outside the surface of the protein is involved in the formation of two cavities in which Ca^2+^ ions are accommodated and plays a key role in the carbohydrate binding. In particular, the principal binding site, constituted by the EPN motif (Glu347, Pro348, and Asn349) together with Glu354 and Asn365 residues, is the one essential for carbohydrate coordination and manages the specificity for the recognized ligands. We therefore hypothesized that the OSR1 could be recognized and linked at the interface of two DC-SIGN monomers, acting as a linker clustering two different tetrameric DC-SIGN units.^21^^,^^38^^,^^39^^,^^40^ With the aim to verify this hypothesis, a new complex composed by two DC-SIGN subunits with the pentasaccharide at the interface between them was modeled.

**Figure 4:**
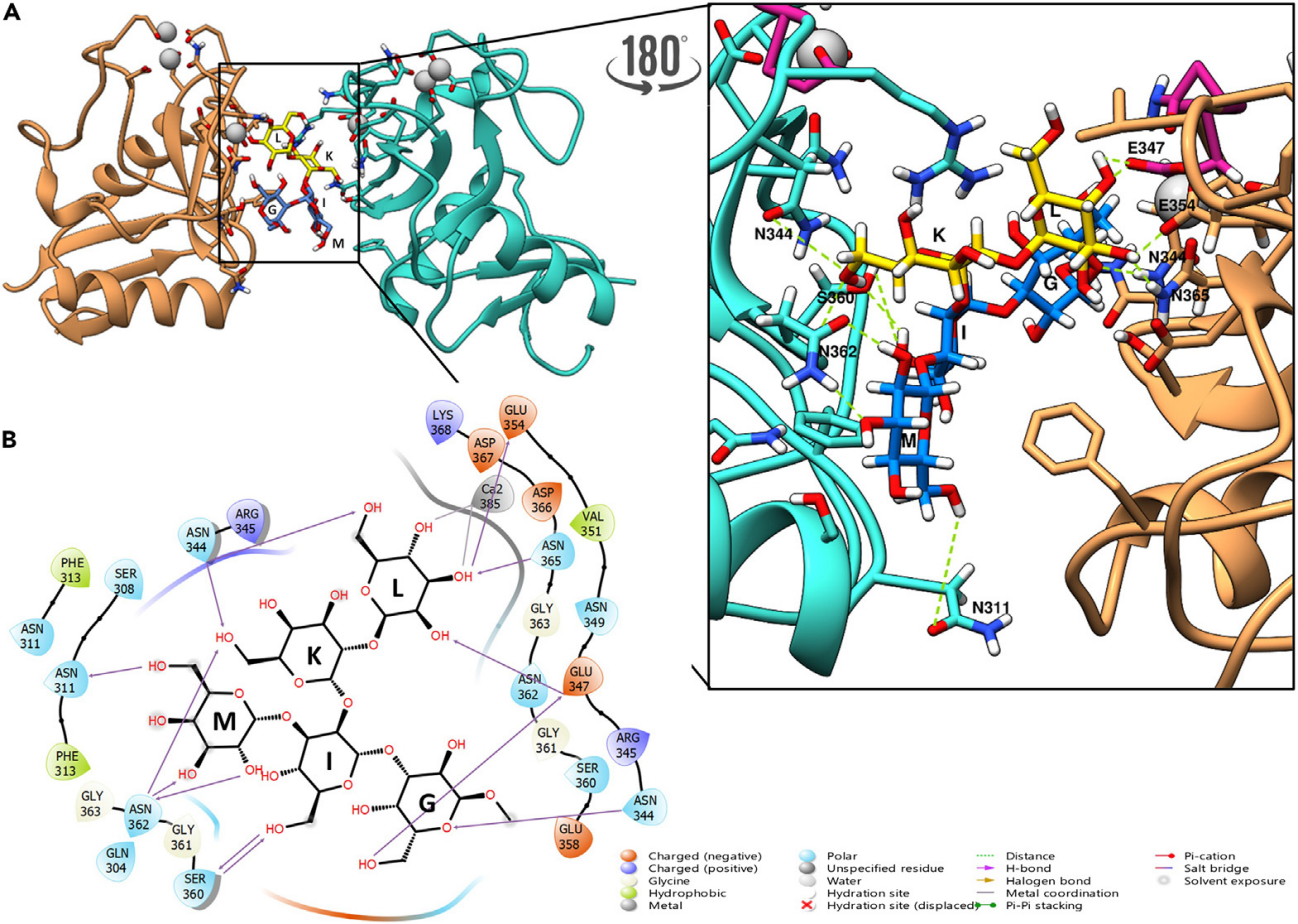
A three-dimensional representative model coming from the 100 ns MD for the complex DC-SIGN dimer—outer core pentasaccharide (A) The ligand is colored with the SNFG color assignment. The outer-core pentasaccharide acts as linker between two different DC-SIGN tetramers. Residues **G** and **L** interact with one subunit of a tetrameric DC-SIGN (orange) while **K**, **I**, and **M** interact with the other subunit of a different tetrameric DC-SIGN (cyan). The interactions between the pentasaccharide and the binding pocket residues are depicted. The most relevant AA involved in the interaction are labeled. The polar contacts are highlighted in green. The EPN motif is highlighted in purple. (B) Two-dimensional schematic plot of the interactions between the DC-SIGN dimer and the outer core pentasaccharide: solid arrows represent the hydrogen bonds with functional groups of the backbone; solid lines represent the Ca^2+^- **L** coordination; the other depicted residues participate with polar and hydrophobic interactions.

**Figure 5:**
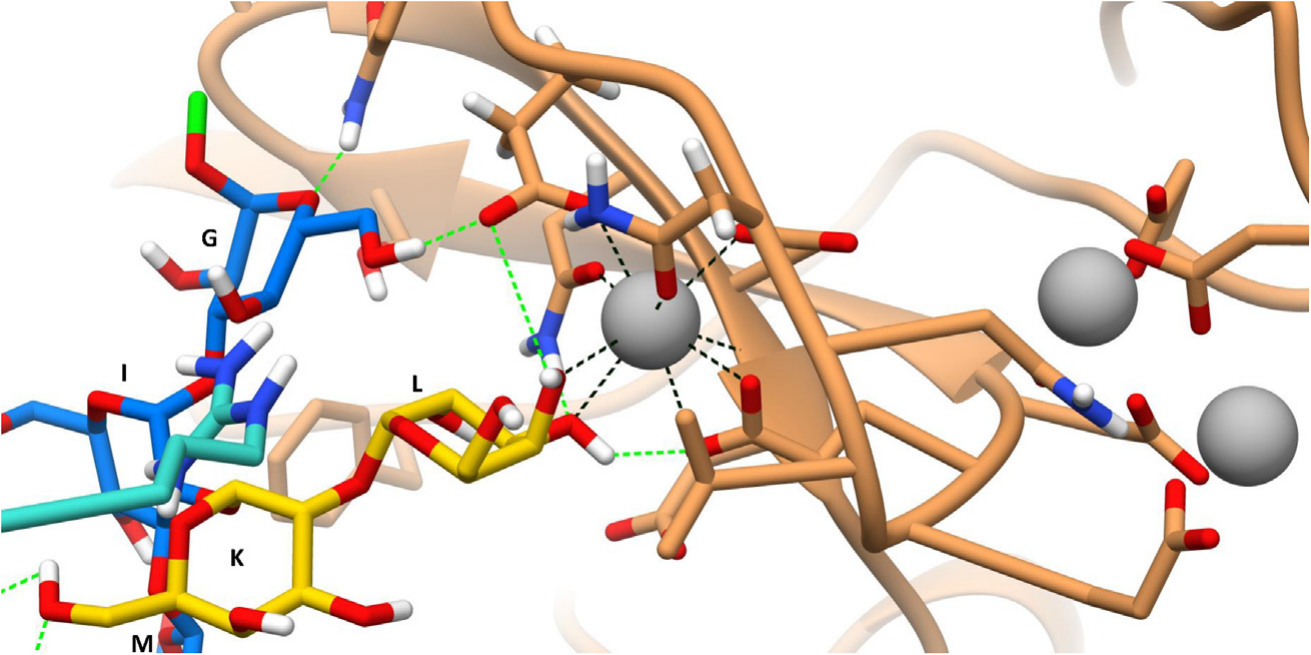
Close up view of the interaction between residues G and L and one of the DC-SIGN subunits The ligand is colored with the SNFG color assignment. The linking point to the rest of OSR1 is depicted in green (residue G). Ca^2+^ ion is colored in gray. The coordination interactions are colored in black while the H-bonds are colored in green.

Interestingly, the complex was stable along the 100 ns trajectory (Figure S6). As shown in Figure 4, where the most representative complex is reported, the binding site of one of the subunits still encloses the ligand, while the other half, solvent-exposed in the monomer, can interact with the second DC-SIGN subunit. In detail, **G** and **L** residues were interacting with DC-SIGN in the principal calcium-dependent binding pocket. The most stable H-bond involved the hydroxyl moiety at position 6 of residue **G** and Glu347. Other H-bonds were instead formed with residue **L**, which was interacting with Glu354 and Asn365 through 3-OH and 4-OH, respectively. Indeed, galactose **L** hydroxyl moieties at positions 3 and 4 were also coordinating with the Ca^2+^ ion (Figure 5). Those interactions were consistent with the literature as recent studies demonstrated the ability of DC-SIGN to recognize α-Galactose^41^ through the coordination of the hydroxyl group at positions 3 and 4 by Ca^2+^. In addition, residues **M**, **I**, and **K** were involved in the interaction with the second subunit. Interestingly, the interaction did not take place in the principal calcium-dependent binding site of this second subunit; those residues were indeed interacting with polar amino acids: Asn311, Asn344, Ser360, and Asn362, which formed stable hydrogen bonds. In detail, 2-OH and 3-OH of residue **M** were stabilized by hydrogen bonds with Asn362, observed for the 73% of the simulation time. This was in agreement with the NMR results given the significant contribution to the interaction of **M** in the NMR studies. Moreover, the residue Asn362 also formed a hydrogen bond interaction with 6-OH of **K**, which remained stable during 43% of the simulation time. The same 6-OH was acting as an H donor with Asn344 for another 53% of the simulation time. As happened with **K**, 6-OH from residue **I** alternated between hydrogen bond donor and acceptor when interacting with Ser360. It is important to highlight that these interactions were the same ones observed in the MD of the cluster B reported before. Therefore, it seems that the coordination and interaction between residues **L** and **G** by itself is not stable enough and is stabilized by the involvement of a polar region of another tetrameric DC-SIGN in the interaction.

Regarding the ligand conformation, the dihedral angles Φ and Ψ have been monitored along the trajectories of the free (data not shown) and bound state (Figures S7 and S8) to evaluate the pentasaccharide conformational behavior before and after the binding to the DC-SIGN dimer. All the dihedral values around the glycosidic linkages were in accordance with the *exo*-anomeric effect (Figure S8), and no significant variations were observed by comparing the free and bound states, thus suggesting that the ligand adopted the same conformation before and upon binding.

**Figure 6:**
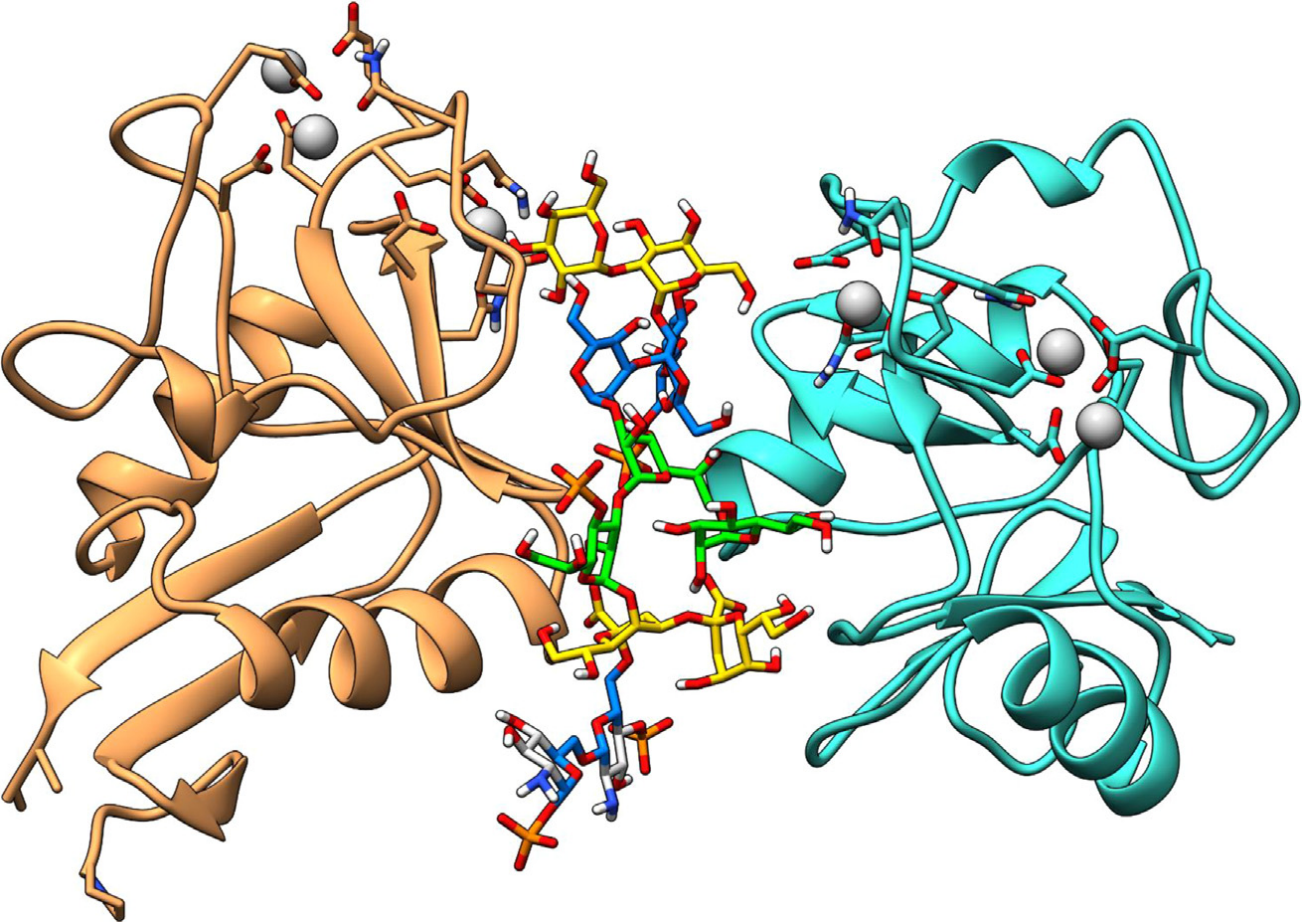
Manual superimposition of the full OSR1 dodecasaccharide with the dimeric DC-SIGN The monosaccharides are colored using the SNFG color code with the phosphate groups in orange. The subunits coming from each tetrameric DC-SIGN unit are colored in sandy brown and turquoise with the calcium ions in gray. It is possible to see how the dodecasaccharide can fit the dimer without crashing the surface of the protein.

**Figure 7:**
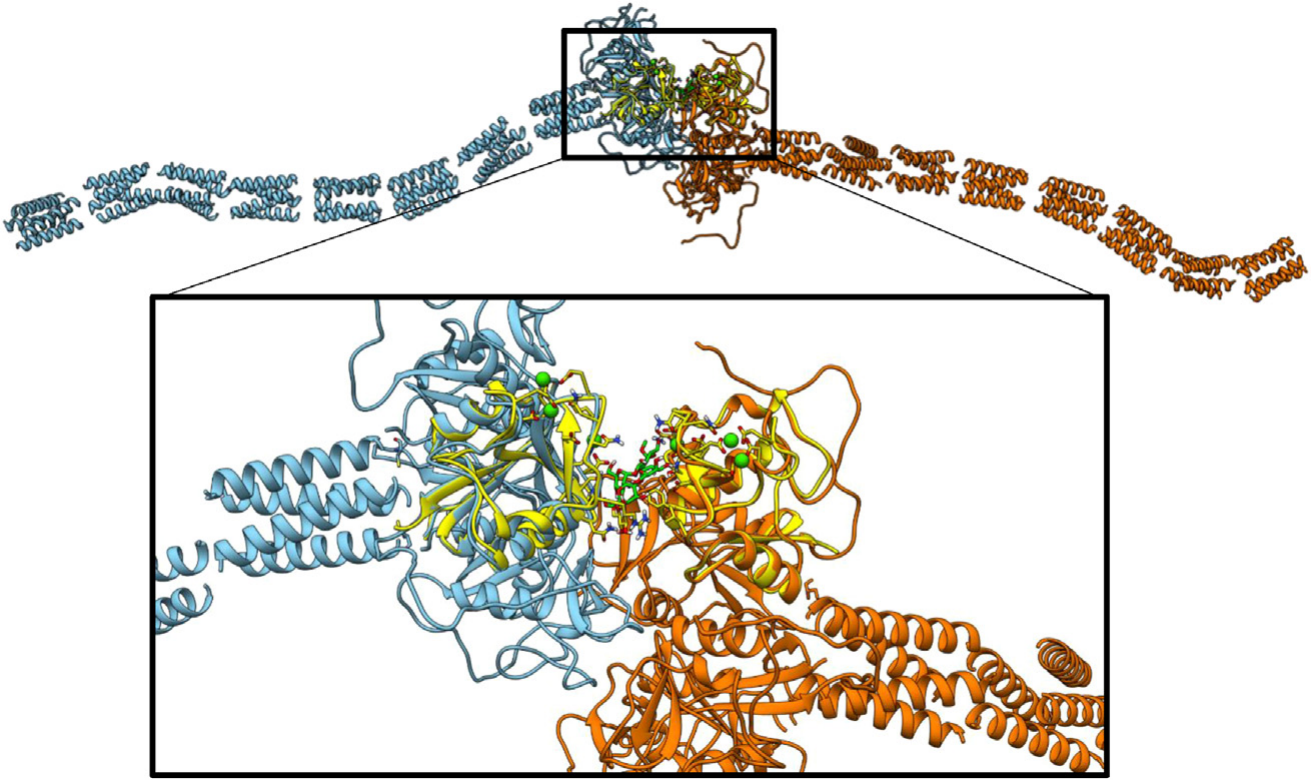
Proposal of the 3D model for the recognition of OSR1 by DC-SIGN with the oligosaccharide acting as crosslinker between two different DC-SIGN tetramers Two full DC-SIGN tetramers with neck (blue and orange) superimposed to the MD pose (yellow). It is possible to observe how the 3D proposed complex in yellow allows the two different DC-SIGN to accommodate without steric hindrances nor any type of clash.

**Figure 8:**
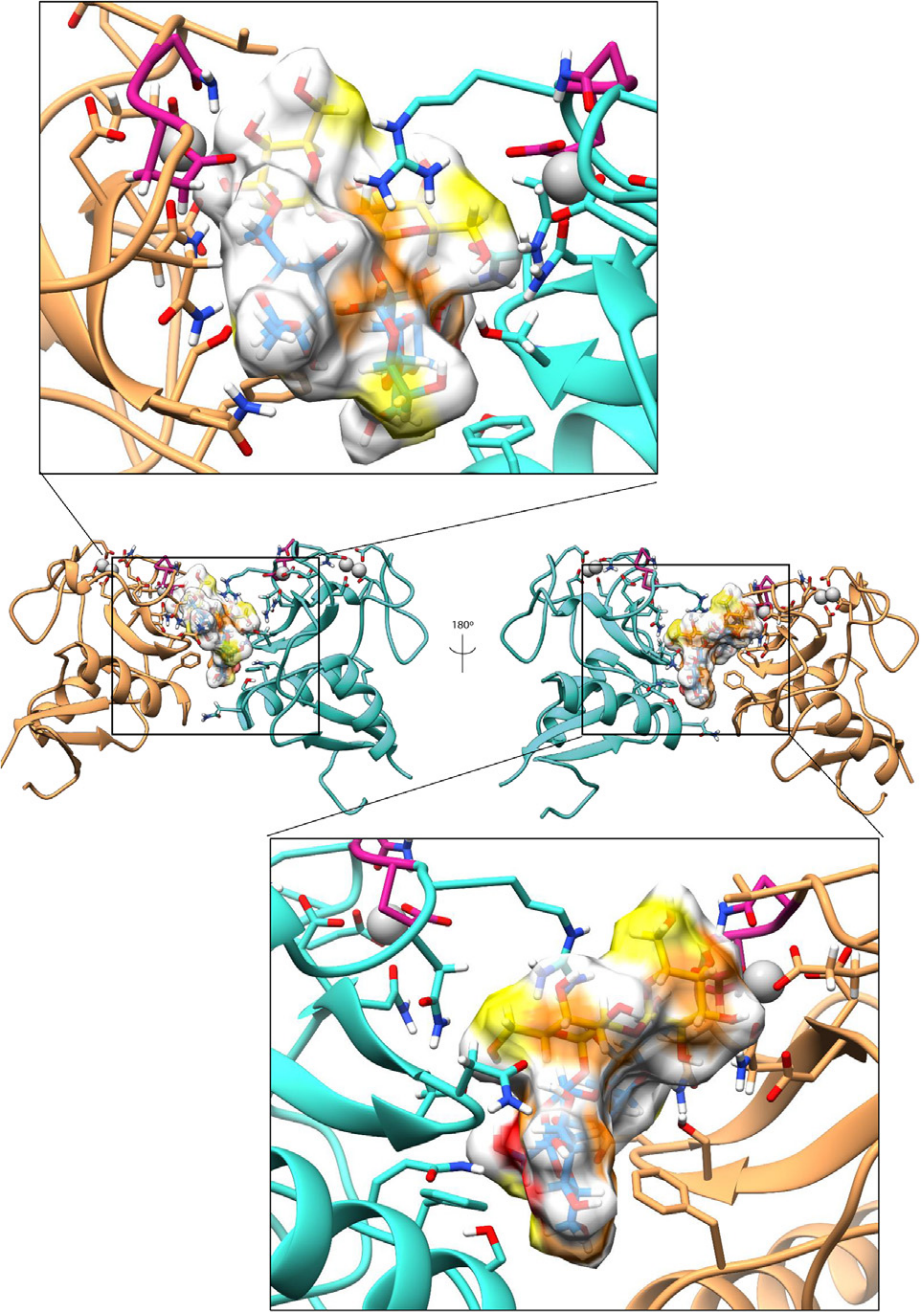
A three-dimensional representative model coming from the 100 ns MD for the complex DC-SIGN dimer—Outer core pentasaccharide The ligand is colored with the SNFG color assignment while its surface is colored according to the STD data. In sandy brown and turquoise there are the subunits coming from each DC-SIGN tetrameric unit. The calcium ions can be found colored in gray.

Potential steric hindrance or clashes when the entire core OS is interacting with the protein were ruled out by modeling the complete OSR1 on one of the representative poses of the MD with the two DC-SIGN units. As showed in Figure 6, the full saccharide can be accommodated in the binding site with the inner core and the lipid A sugars solvent exposed, without clashing with the protein. Moreover, the two DC-SIGN tetramers were superimposed to the system to discard clashes between protein chains; as can be seen in Figure 7, there were neither clashes nor steric hindrance that could affect the proposed complex. The observed results were consistent with NMR studies, as the interaction between the second subunit and saccharides **M**, **I**, and **K**, could justify the presence of STD NMR enhancements in all the pentasaccharide (Figure 8).

## Discussion

Given the pivotal roles that lectins play in cellular trafficking and cell-cell communication they have been widely recognized as therapeutic targets. Worthy of note is the role played by DC-SIGN (dendritic cell-specific intracellular adhesion molecules (ICAM)-3 grabbing non-integrin), in the regulation of the immune response upon the recognition of carbohydrate structures exposed on pathogens or self-glycoproteins. In particular, this C-type lectin exhibits several functions including adhesion, migration, signaling, and antigen uptake/presentation. Although our understanding of the biological roles of DC-SIGN has been expanding during the last years, a detailed molecular knowledge of its interaction with bacterial glycan ligands is still not fully disclosed.

In this *scenario*, we here demonstrated the ability of DC-SIGN to recognize R1-cells core OS by an integrated approach based on fluorescence microscopy, SPR, NMR, and computational studies. Fluorescence microscopy primarily allowed to assess the ability of DC-SIGN ECD to strongly bind *E. coli* surface presenting R1 type core oligosaccharides; SPR provided an estimation of the affinity values; the ligand conformation and epitope mapping was profiled by combining the results achieved by STD NMR and computational studies allowing to propose a 3D model of the interaction.

Overall, the results achieved on DC-SIGN ECD in the presence of the isolated OSR1 showed that the binding process mainly involved the carbohydrate residues of the outer core, with an IC_50_ of 1 mM, suggesting a large epitope of interaction. In detail, we here proposed a 3D model in which **G** and **L** OS units (Figure 2A) were interacting with DC-SIGN in the principal calcium-dependent binding pocket, with the galactose residue **L** coordinating with the Ca^2+^ ion through the hydroxyl groups at positions 3 and 4. The binding is further strengthened by the interaction of the ligand with a second subunit of the protein, mediated by residues **M**, **I**, and **K**, which interestingly seemed to be accommodated in a calcium-independent secondary binding-site. Notably, the presence of additional secondary binding sites on DC-SIGN CRDs assembled in tetramers, far from the primary Ca^2+^ dependent pocket and able to increase the affinity for multivalent ligands, has been previously hypothesized.^32^ In such a DC-SIGN ECD dimer, it is also possible that additional weak interactions are promoted between each DC-SIGN tetramer near the OSR1 binding interface. Such additional stabilizing interactions cannot be observed by STD NMR but cannot be excluded.

According to our three-dimensional model, the CRDs orientation of the DC-SIGN ECD allows the recognition of OSR1 at the interface of two different tetrameric units of the protein. We demonstrated the main engagement of the outer core region in the recognition and interaction process that involves not only the primary calcium-dependent binding pocket but also a secondary binding site positioned on a second subunit of the protein. One could argue that the molecular complex depicted here is just possible in the conditions used in the NMR experiment where both DC-SIGN ECD and the OSR1 OS are free in solution. We cannot exclude the possibility of a different mode of interaction in the physiological conditions where DC-SIGN ECD is embedded in a cell membrane and the core OS is presented on LOSs inserted in the *E. coli* outer membrane. However, both the epifluorescence and STORM microscopies on one side, where R1-cells LOS is presented in the physiological context of the outer membrane, and the SPR interaction test on the other side, using oriented surfaces of DC-SIGN interacting with LOS embedded within micelles, confirmed the relevance of the DC-SIGN/OSR1 interaction. Both experiments better mimics physiological conditions. The different presentation of the two partners of the interaction may generate avidity and potential multiple interactions through clustering and/or chelating mode,^42^ as also suggested by the higher affinity in the SPR direct interaction test. Such multivalent interaction mode could be generated, instead of an OSR1 pentasaccharide sandwiched between two DC-SIGN tetramers as seen here, by a combination of independent DC-SIGN CRDs binding with several LPSs and exploiting different combination of the two possible interacting interfaces (with residue **G** and **L** on one side or residues **I, K**, and **M** on the other) highlighted here thanks to the STD NMR experiments. Indeed, a DC-SIGN tetramer could establish, using its different CRDs, multiple multivalent binding modes with the *E. coli* outer membrane surface considering that these cumulative binding will be mutually self-stabilizing. To note, we have already observed such multivalent interaction between another C-type lectin, MGL, and R1-cells LOS.^43^

The outcomes showed here provided fine atomic structural details for the interaction occurring between DC-SIGN and microbial glycans. It should be noted that, given the high homology in the sequences of many genes involved in the synthesis of LPS among the members of the family of Enterobacteriaceae, our results suggest that DC-SIGN may also serve as a receptor for other bacterial strains. As example, *E. coli*, *Shigella dysenteriae* and other Enterobacteriaceae are very closely related genetically and their core OS structures are very similar,^44^ thus, it would not be surprising finding out the ability of DC-SIGN to recognize and bind also *Shigella* through the interaction with its core OS.

### Limitations of the study

The present study describes the interaction between human DC-SIGN and the purified deacylated R1 type lipooligosaccharide of *E. coli* through a combination of complementary techniques, including fluorescence microscopy, SPR, NMR spectroscopy, and computational studies.

Given the acknowledged limitations of docking for systems with weak affinity, such as the one addressed here, these calculations were only used to derive the initial complex for MD simulations. The MD outcomes revealed that the binding between DC-SIGN and *E. coli* LOS occurs through the recognition of the outer core pentasaccharide that can act as a crosslinker between two distinct DC-SIGN tetramers. To further validate the proposed recognition model involving DC-SIGN dimers, we conducted preliminary AUC studies that look promising (data not shown), however, additional experiments are needed. Further development of this work may also include the investigation of DC-SIGN’s potential to identify and bind additional gram-negative bacteria belonging to the *Enterobacteriaceae* family through interactions with their core OS.

## STAR★Methods

### Key resources table

**Table:** 

REAGENT or RESOURCE	SOURCE	IDENTIFIER
**Bacterial and virus strains**		

E. coli F470 (derivative from E. coli O8:K27)	S. Muller-Loennies	N/A
E. coli BL21(DE3)	Sigma-Aldrich	CMC0014

**Chemicals, peptides, and recombinant proteins**		

BSA-Manα1–3[Manα1–6]Man	Dextra Laboratories	NGP1336
Streptavidin	Sigma-Aldrich	S4762
Biotin-LPET(depsi)GG	GENECUST	Custom peptide.

**Recombinant DNA**		

pET-30b	Novagen	69910
pET-28a	Novagen	69864

**Software and algorithms**		

MACS quant software	Milteny Biotech	N/A
Image J	Fiji (imagej.net)	N/A
AMBER18	https://ambermd.org/	N/A
Imaris	Oxford Instruments	N/A
Biacore T200 Evaluation Software 3.2.1	GE Healthcare	N/A

### Resource availability

#### Lead contact

Further information and requests for resources and reagents should be directed to and will be fulfilled by the lead contact, Professor Roberta Marchetti (roberta.marchetti@unina.it).

#### Materials availability

This study did not generate or used new unique reagents.

#### Data and code availability

Every numerical data in this study is included in Supplementary Data.

The code used for the Stoddart diagram and dihedral analysis is available from the lead contact upon request.

Any additional information required to reanalyze the data reported in this paper is available from the lead contact upon request.

### Experimental model and study participant details

This study did not include experiment with specific model or subject.

### Method details

#### Fluorescence microscopy

DC-SIGN ECD was labelled with Alexafluor647-NHS(Invitrogen). Briefly DC-SIGN at 5 mg/ml in PBS buffer was incubated in 200mM sodium bicarbonate and 0.4 mg/ml AF647-NHS for one hour. Excess dye was removed with G25-PD10 desalting column (GE Healthcare) and DC-SIGN fractions dialyzed further against PBS buffer and concentrated. *E. coli* bacteria carrying R1 type core oligosaccharide (F470 derivative from *E. coli* O8:K27 were grown in LB at 37°C under agitation up to 0.9 OD600nm. Cells were collected by centrifugation, washed in cold PBS and incubated with 670 nM DC-SIGN AF647 in PBS, 2mM CaCl2 buffer for 15 min. Cells were washed five times with cold PBS and imaged.

For epifluorescence microscopy, 2 μL of cells in suspension were mounted between a glass slide and a 170 μm glass coverslip (1.5H). The sample were observed with an inverted IX83 microscope using a UPLFLN 100× oil immersion objective from Olympus (numerical aperture 1.49), a fibered CoolLED PE-4000 excitation LED at 635nm, in combination with the appropriate excitation filters, dichroic mirrors, and emission filters (LED-DA/FI/TR/Cy5/Cy7-A set and AF647 specific FF01-684/24 emission filter, Semrock). Acquisitions were performed with Volocity software (Quorum Technologies) using a sCMOS 2048 × 2048 pixels camera (Hamamatsu ORCA Flash 4, 16 bits/pixel) achieving a final magnification of 64 nm per pixel.

For 3D super-resolution dSTORM (direct STochastic Optical Reconstruction Microsopy) imaging, *E. coli* cells labelled with DC-SIGN-Alexa647 as described above were applied on a coverslip, and allowed to settle for 2 minutes. Supernatant was removed and replaced by a glucose buffer containing 1.5 % low melting agarose, 50 mM Tris, 10 mM NaCl (pH 8.0), 10% glucose, 100 mM MEA (mercaptoethylamine), 0.34 mg/ml catalase and 5.6 mg/ml glucose oxidase, and immediately covered by a glass slide. The 3D astigmatism acquisitions were performed on a Abbelight™ SAFe360 / Olympus IX83 setup equipped with an anti-drift ZDC2 at 830 nm, and UPLAPO100XOHR (NA 1.5) 100x oil immersion objective. Excitation with a fibered laser source (Oxxius L6CC combiner) was used to generate the AF647 blinking regime. Illumination in HiLo mode provides a uniform excitation field. We used a combination of 643 nm Laser (at typically 1kW.cm-2), in the increasing presence of low power density 405 nm laser (up to 3.10-1W.cm-2) to maintain the number of detected spots per frame. The acquisitions were performed in parallel (50/50 mirror splitter) on 2 ORCA Fusion sCMOS (2304 × 2304 pixels; 16bits/píxel – Hamamatsu) at 50ms exposure time for 35000 frames using a limited 308 × 308 subarray. The final pixel size is 100nm. The signal is collected directly or through a spherical lens to generate astigmatism. The 2D widefield epifluorescence image is the maximum intensity projection of the acquired stack of the direct camera, whereas the super-resolution STORM image is generated after analysis using weighted least square localisation algorythm (Thunderstorm plugin^45^ – Fiji^46^). Localisation files have been further processed in Fiji for filtering and Imaris (Bitplane) for 3D rendering and overlays.

#### Production and purification of recombinant proteins

##### DC-SIGN ECD production

Plasmid pET-30b (Novagen) containing cDNA encoding the Extra Cellular Domain (ECD) (corresponding to amino acids 66–404) of DC-SIGN, cloned between Nde I and BamH I was used for 1 mM IPTG-induced overproduction as inclusion bodies at 37°C into BL21(DE3) cells.^47^ Cells were lysed by sonication into buffer A (25 mM TRIS-HCl pH 8.0, 150 mM NaCl and 4 mM CaCl_2_) and inclusion bodies were isolated by a 30 min centrifugation at 100,000 g. Two resuspension / centrifugation cycles were done to wash inclusion bodies into buffer A supplemented by 2 M urea and 1% v/v X-100 Triton and in buffer A. Inclusion bodies were finally solubilized in 30 mL/L of culture of buffer A supplemented by 6 M guanidine/HCl and 0.01% v/v β-mercapto-ethanol. The supernatant of a 30 min centrifugation at 100,000 g, diluted at 2 mg/mL (concentration based on DC-SIGN ECD ε) was used for refolding by a five-fold flash-dilution into a buffer containing 25 mM TRIS pH 8.0, 1.25 M NaCl, and 25 mM CaCl_2_ and followed by 3 dialysis steps of 3 h. First dialysis was done into 7 volumes of water and the two others into buffer A.^48^ Purification of functional DC-SIGN ECD was achieved by an affinity chromatography on mannan-agarose column (Sigma) equilibrated in buffer A, and eluted in the same buffer lacking CaCl_2_ but supplemented with 1 mM EDTA. This step was followed by a Superose 12 size exclusion chromatography equilibrated in buffer A.^49^ Final protein sample was concentrated to 10 mg/mL.

DC-SIGN ECD was dialyzed three times against the deuterated buffer 25 mM TRIS DCl, 150 mM NaCl, 4 mM CaCl_2_ at pD 7.8 in D_2_O (deuterated TRIS-d11 (98%) was purchased from Cambridge Laboratories Inc. and the D_2_O from Spectra Stable Isotopes). Protein was flash frozen and stored in liquid nitrogen.

##### DC-SIGN Biot-ECD production

To produce DC-SIGN Biot-ECD, the same protocol as for DC-SIGN ECD was used with a plasmid containing the same sequence with addition of 3 glycines at N-terminus of the protein. These 3 glycines were used to label DC-SIGN ECD with a synthetic biotin-LPET(depsi)GG peptide (GENECUST) using sortase A from *Staphylococcus aureus*. DC-SIGN 3Gly-ECD (1 molar equivalent) was mixed with biotin-LPET(depsi)GG peptide (1.5 molar equivalent) and sortase A (0.2 molar equivalent) from *Staphylococcus aureus*, recombinantly produced in the laboratory, in 25 mM TRIS-HCl pH 8, 150 mM NaCl, 4 mM CaCl_2_ buffer. The reaction was incubated at 37°C for 6 h under agitation and reaction product was purified on a Superose 12 size exclusion chromatography equilibrated in buffer A. Final protein sample was concentrated to 1 mg/mL.

##### DC-SIGN CRD production

To produce DC-SIGN CRD, the same protocol as for DC-SIGN ECD was used with a plasmid containing cDNA encoding the Carbohydrate Recognition Domain (CRD) (corresponding to amino acids 250–404) of DC-SIGN with addition of a 6 His-Tag and a factor Xa cleavage site at N-terminus, cloned between Nde I and BamH I restriction site. ^15^N-labelled DC-SIGN CRD was produced in M9 minimal medium with ^15^NH_4_Cl as sole nitrogen source.^43^ The product of dialysis after refolding was purified by an affinity chromatography on HisTrap HP column (GE Healthcare) equilibrated in buffer A, and eluted in the same buffer supplemented with 500 mM imidazole. This step was followed by a Toyopearl HW-50 S (Tosoh Bioscience) size exclusion chromatography equilibrated in buffer A. Final protein sample was concentrated to 50 μM.

##### Sortase A production

To produce sortase A, plasmid pET-28a (Novagen) containing cDNA encoding the amino acids 26–206 of *Staphylococcus aureus* sortase A (Q9S446, UniProt) with addition of MGSSHHHHHHSSGLVPRGSH sequence (6-His Tag, thrombin cleavage site and cloning residues) at N-terminus, cloned between Nco I and EcoR I was used for 1 mM IPTG-induced overproduction of protein. Cells were lysed by sonication into 25 mM TRIS-HCl pH 8.0, 150 mM NaCl buffer and the supernatant of a 30 min centrifugation at 100,000 g was used for purification by an affinity chromatography on HisTrap HP column (GE Healthcare) equilibrated in lysis buffer, and eluted in the same buffer lacking supplemented with 500 mM imidazole. This step was followed by a Superdex 75 size exclusion chromatography equilibrated in lysis buffer. Final protein sample was concentrated to 1 mM.

#### Flow cytometry

Flow cytometry was recorded on a VYB device (Miltenyi biotech) and analyzed with Macsquant software. 50 μl of F470 Cells grown in LB at OD600nm=0.8 were washed once in PBS, then resuspended in presence of 670 nM DC-SIGN-AF647 in PBS, 2 mM CaCl_2_ with/without 1 or 2 mM OSR1. The samples were incubated for 15 min at room temperature in the dark, washed twice to remove excess protein, then resuspended in 250 μl PBS and injected for FACS analysis (total 200000 events recorded). DC-SIGN-AF647 binding to cells was expressed as % population x median fluorescence (cy5 channel) and normalized to 100 % for DC-SIGN binding in absence of OSR1. Experiments were performed as triplicates.

#### LOS R1 extraction and purification

*E. coli* F470 (R1) was extracted following the Phenol/Chloroform/light Petroleum (PCP) method. The dry cells were mixed with a 2.5% PCP solution, stirred, and centrifuged to collect supernatant. The pellet underwent a repeated treatment, while the supernatants were saved. The supernatant was processed to remove solvents, leaving phenol and water traces. The phenol solution was mixed with water until LOS precipitated. After centrifugation, the LOS precipitate was washed, dried, suspended in water, and freeze-dried. The phenol supernatant was diluted, dialyzed, and freeze-dried.

A portion of pure LPS underwent treatment with anhydrous hydrazine (2 mL), followed by stirring at 37°C for 90 minutes. The resulting mixture was then cooled, poured into ice-cold acetone (20 mL), and left to precipitate. After centrifugation (4000g, 30 minutes), the precipitate was washed with ice-cold acetone, dried, dissolved in water, and subjected to lyophilization. Subsequently, the O-deacylated product underwent N-deacylation with 4 M KOH. To eliminate salts, gel-filtration chromatography on a Sephadex G-10 column (Pharmacia, 50 × 1.5 cm) was employed. The fully deacylated product underwent further purification using a Toyopearl TSK HW-40 column (Tosoh Bioscience). More information of the protocols used can be found in *De Castro,* et al. work.^50^

#### LOS R1 micelles preparation

DDM-LOS R1 micelles were prepared after adding 150 mM of DDM to purified LOS vesicles of R1 at 0.84 mM in HEPES Buffered Saline buffer (10 mM HEPES 150 mM NaCl 2 mM CaCl2 pH 7.4). The mixture was kept under gentle rocking for 15 min at RT. Insoluble material was removed by ultracentrifugation at 100 000 g for 30 min and the sample homogeneity assessed by Dynamic Light Scattering.

#### Surface plasmon resonance (SPR) analysis

All experiments were performed on a Biacore T200 using functionalized CM3 sensor chips. Competition experiments were performed using flow-cells functionalized with mannosylated-BSA. Flow cells were activated as previously described.^51^ Flow cell 1 was functionalized with final density of 2112 RU of BSA and used as a control surface, flow cell 2 and 3 were functionalized with final density of 1960 RU and 2067 RU of BSA-Manα1–3[Manα1–6]Man (BSA-Mannotriose, Dextra), respectively. The BSA-Mannotriose used to functionalize sensor chip harbors an average coupling ratio of 13 α1-3, α1-6 Mannotriose per BSA with 14 atoms spacer. The affinity of OSR1 was then evaluated through determination of its IC50 using a DC-SIGN ECD binding inhibition assay. The ECD of DC-SIGN was injected onto the BSA-Mannotriose surface, at 20 μM alone or in presence of an increasing concentration of OSR1, from 5 μM to 5 mM. Injections were performed at 30 μL/min using 25 mM Tris-HCl, pH 8, 150 mM NaCl, 4 mM CaCl2, and 0.05% of P20 surfactant as running buffer. Analysis has been performed using the steady-state interaction model. The Req steady state binding responses of DC-SIGN ECD to BSA-Mannotriose surface were obtained from sensorgrams of each conditions and converted to relative residual activity values. Relative IC50 values were determined from the plots of relative residual DC-SIGN ECD activity vs OSR1 concentration and fitted using four-parameter logistic model (Equation 1); where Rhi and Rlo are maximum and minimum asymptotes of activity, A1 is the inflection point and A2 is a slope of the curve. To end, IC50 was calculated using Equation 2:

(Equation 1) $$y=R_{hi}-\frac{R_{hi}-R_{lo}}{1+{\left(\frac{Conc}{A_{1}}\right)}^{A_{2}}}$$

(Equation 2) $${IC}_{50}=A_{1}\cdot {\left(\frac{R_{hi}-R_{lo}}{R_{hi}-50}-1\right)}^{\frac{1}{A_{2}}}$$

SPR Direct interaction analysis using oriented surfaces of DC-SIGN have been done using a DC-SIGN ECD construct that was specifically biotinylated on its N-terminus (here after called DC-SIGN Biot-ECD). This construct is overexpressed and purified according to previously published protocol.^52^ Streptavidin (Ref.: S4762; SIGMA-ALDRICH) diluted at 100 μg/mL in 10 mM NaOAc pH 4 was immobilized on sensor chips sensor chip S Serie CM3 (Cytiva). DC-SIGN Biot-ECD was captured onto the streptavidin functionalized surfaces. This Biot-ECD version of DC-SIGN-ECD results from a site directed N-terminal biotinylation that allow a uniform orientation of the CLRs ECD on the surface mimicking the natural presentation at the cell surface. This site specific biotinylation on the N-termini are performed in house thanks to a sortagging procedure previously described in Achilli et al.^53^. DC-SIGN Biot-ECD was diluted respectively at 0.5 μg/mL in running buffer (HBS-N, 2 mM CaCl2 and 300 μM DDM buffer), injected at 5 μL/min until to a capture level around 1264.4 RU was achieved. For interaction measurements, R1-cells LOS were prepared as described above and were injected at concentrations ranging from 2.6 μM to 85 μM over DC-SIGN Biot-ECD oriented surface using running buffer at 20 μL/min. Streptavidin flow cell surface was used as reference for correction of the binding response. Regeneration of the surfaces was achieved by 50 mM EDTA, pH 8. Binding curves were analyzed using Biacore T200 Evaluation Software 3.2.1 (GE Healthcare) and data were fit using Steady State Affinity model.

#### NMR analysis

The NMR experiments were recorded on a Bruker AVANCE NEO 600 MHz equipped with a cryo probe. Samples were prepared in buffer (Tris 25 mM, CaCl2 4 mM, NaCl 150 mM, pH = 8) and 2,2,3,3-d(4)-3-(trimethylsilyl)propionic acid sodium salt (TPS 10 μM) was used as the internal reference for the spectra calibration. STD NMR experiments were acquired on a protein:ligand mixture with a molar ratio of 1:50, by using 32 k data points and zero-filled up to 64 k data points prior to processing. The protein resonances were selectively irradiated using 40 Gauss pulses with a length of 50 ms, setting the off-resonance pulse frequency at 40 ppm and the on-resonance pulse at 0 and 7.5 ppm.

Human 15N labelled DC-SIGN CRD domain at 50μM in 25 mM Tris pH 8, 150 mM NaCl, 4 mM CaCl2 was titrated with increasing concentrations of OSR1 up to 20 molar equivalents OSR1:CRD. 1H-15N Best-Trosy experiments were recorded at 37°C on an 850MHz Bruker NMR spectrometer equipped with a cryoprobe at each oligosaccharide addition. Assignment of 1H,15N resonances deposited in bmrb n°27854^41^ were used to analyze the titrations with CccpNmr analysis v3 software.

#### Docking calculations

Docking calculations were performed using AutoDock 4.2.2 and analysed with AutoDockTools.^36^ The ligand was downloaded from GLYCAM website (www.glycam.org)^35^ and all rotable bonds were set as free to move during calculations with AutoDockTools. Analysis of the docking poses was performed with AutoDockTools. The grid point spacing was set at 0.375 Å, and a hexahedral box was built with x, y, z dimensions: 40–80 Å, 40–80 Å, 40–80 Å centered in the centroid position among DC-SIGN binding pocket residues. A total of 200 runs using Lamarckian Genetic algorithm were performed, with a population size of 150, and the maximum number of energy evaluations set at 2500000. After docking, the 200 poses were clustered in groups with root-mean-square deviation less than 2.0 Å and the clusters ranked according to the lowest energy representative of each cluster.

#### Molecular mechanics and molecular dynamics simulation

Molecular mechanics calculations were performed using the MM3∗ force field in the vacuum with a dielectric constant of 80 was used. Disaccharide structures were explored by incrementally varying both Φ and Ψ at a grid step of 18°. Each (Φ, Ψ) point of the map was optimized using 2000 P.R. Molecular dynamic calculations were performed with AMBER 18 software^54^ in explicit waters using AMBER ff14SB, Glycam06j-1 and TIP3P force fields for the protein residues, the saccharide ligand and the water solvent molecules respectively. The different ligand were downloaded from GLYCAM website (www.glycam.org).^35^

To prepare the protein, missing hydrogen atoms were added, and protonation state of ionisable groups and cap termini was computed using Maestro Protein Preparation Wizard.^55^ The systems followed the same protocol, being hydrated with an octahedral box containing the explicit TIP3P water molecules buffered at 15 Å, with the addition of counterions to neutralize the system. Input files were generated using the tLeap modules of the AMBER package. The Sander module was used for the minimization steps while molecular dynamic calculations were performed using the PMEMD module. At this point, an energy minimization process was performed to refine the initial structure. The calculations employed SHAKE for the C-H bonds and 1 fs of integration step.

Periodic boundary conditions were applied with the smooth particle mesh Ewald method to represent the electrostatic interactions, with a grid space of 1 Å. At first the system was minimized holding the complex, while a further minimization step was performed on the entire system. The system underwent a gradual heating process from 0 to 300 K, with a weak restraint on the solute. Temperature increased from 0 K to 100 K at constant volume and then from 100 K to 300 K in an isobaric ensemble. Subsequently, temperature was maintained at 300 K for 50 ps with progressive energy minimizations and solute restraint. After equilibration, restraints were removed, and the systems advanced in an isothermal-isobaric ensemble during production.

The system coordinates were saved and used for the 100 ns simulations using the PMEMD module implemented in AMBER. Coordinate trajectories were recorded each 2 ps throughout production runs, yielding an ensemble of 10000 structures for each complex, which were finally analysed.

Trajectories were analysed using the ptraj module in AMBER 18, and VMD was used for visualization.^56^ Cluster analysis with respect to ligand RMSD was performed using the K-mean algorithm implemented in the ptraj module. The representative structure of the most populated cluster depicted the complexes' interactions. Hydrogen bond determination utilized the CPPTAJ module in AMBER 18,^57^ defining a bond between an acceptor heavy atom A, a donor hydrogen atom H, and a donor heavy atom D, with distance and angle cutoffs set at 3 Å and 135°, respectively. 3D images were prepared using the USCF Chimera program.^58^ Dihedral conformation analysis utilized a custom script to illustrate torsion variation during MD simulations and generate a histogram of the most populated values.
