# Supplementary material for: Molecular recognition of Escherichia coli R1-type core lipooligosaccharide by DC-SIGN
Source: iScience. 2024 Jan 4;27(2):108792. doi: 10.1016/j.isci.2024.108792 (PMC10828809; doi:10.1016/j.isci.2024.108792)
Supplement: Document S1. Figures S1–S8 [file mmc1.pdf]

iScience, Volume *27*

Supplemental information

Molecular recognition of *Escherichia coli* R1-type core lipooligosaccharide by DC-SIGN

Ferran Nieto-Fabregat, Angela Marseglia, Michel Thépaut, Jean-Philippe Kleman, Massilia Abbas, Aline Le Roy, Christine Ebel, Meriem Maalej, Jean-Pierre Simorre, Cedric Laguri, Antonio Molinaro, Alba Silipo, Franck Fieschi, and Roberta Marchetti

Supporting Information

Table of Contents

Supporting figures **S2-S6**

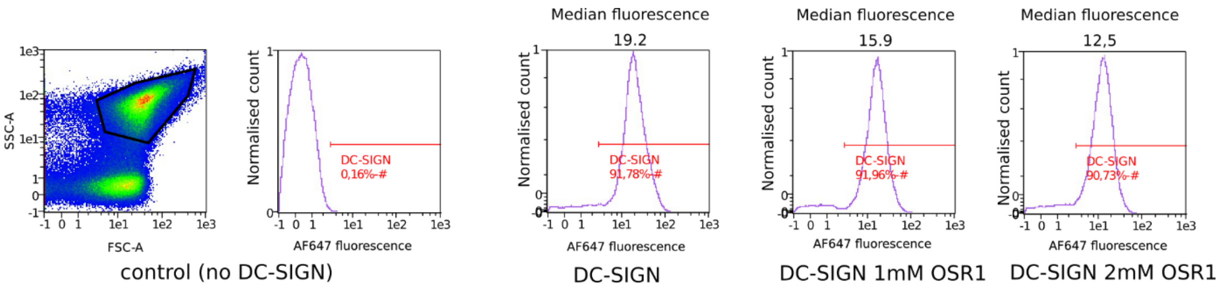

Figure S1: Flow cytometry analysis. Related to Figure 1. Flow Cytometry of F470 cells with/without DC-SIGN-AF647 in absence/presence of OSR1. Left selection of bacterial population on 2D representation of Forward (FSC) and side (SSC) scattering and on the right count vs fluorescence of the selected bacterial population. Only the population within the red range was considered to have significant fluorescence. Associated % of population is indicated as well as the value of median fluorescence.

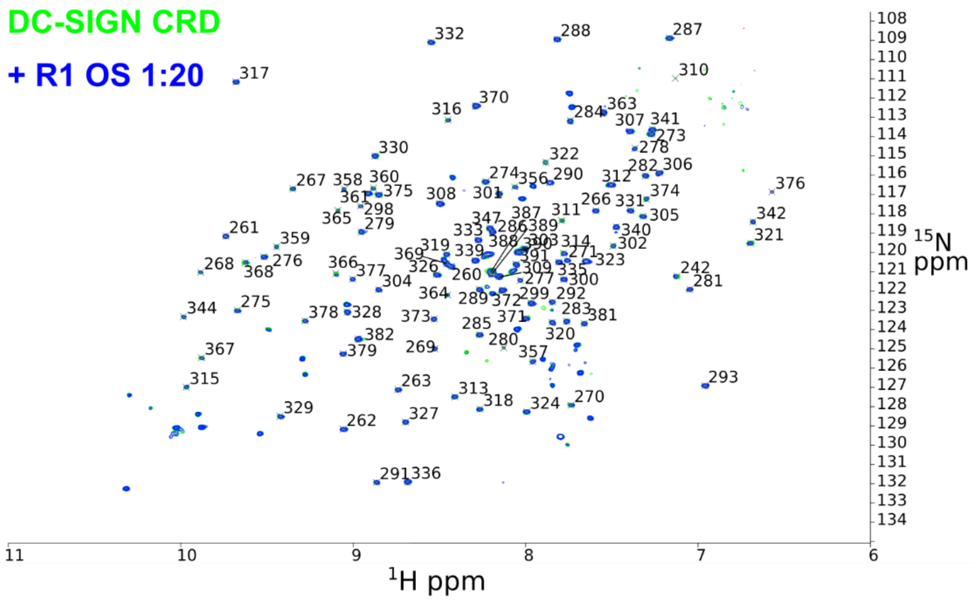

Figure S2: DC-SIGN CRD displays no chemical shift perturbation in presence of OS R1 suggesting very low affinity. Related to Figure 2. ^{1}H-^{15}N Best-Trosy spectra of DC-SIGN CRD without (green) and with (blue) 20 molar equivalents of OSR1.

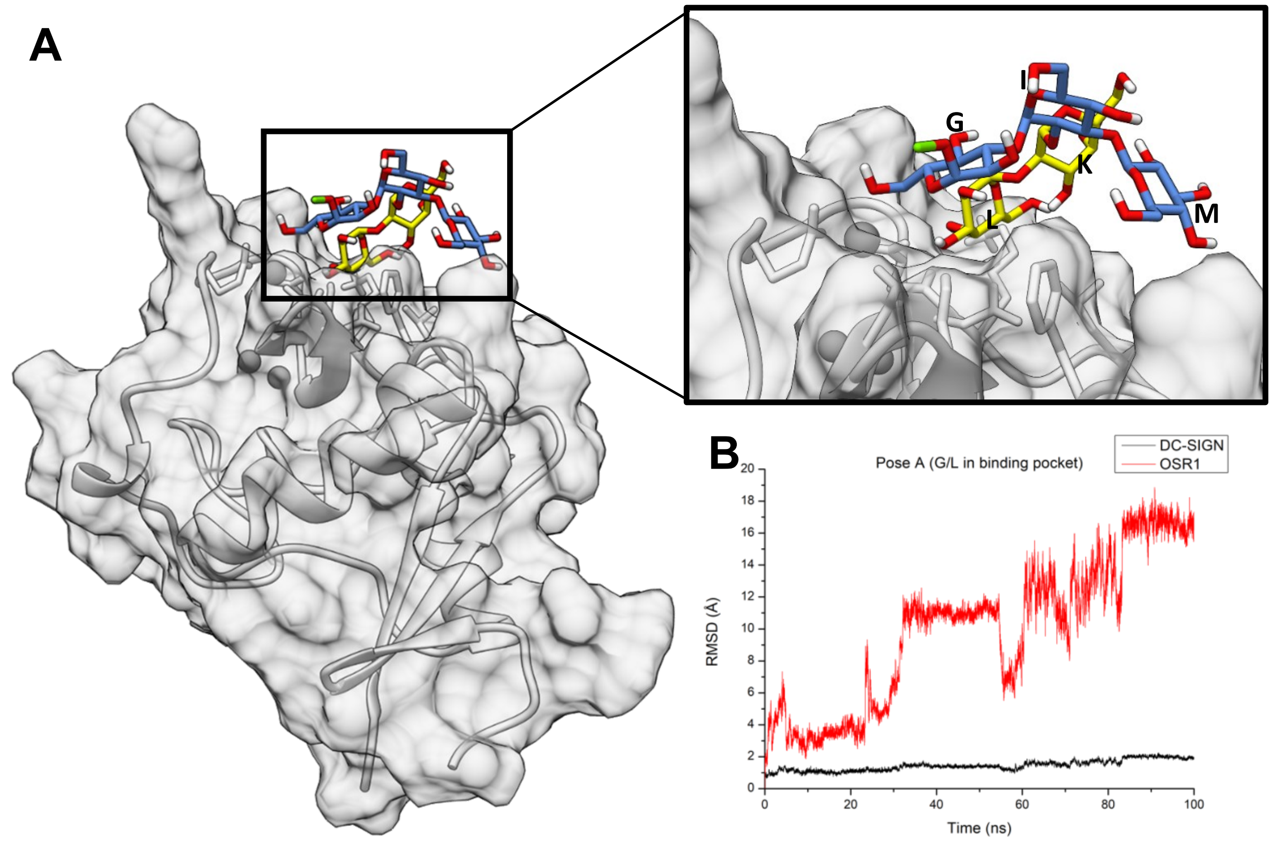

Figure S3. Less energetic cluster (cluster A) from the docking studies between DC-SIGN monomer and OSR1 outer core pentasaccharide. Related to Figure 4. A) Representative pose of the so-called cluster A result of the docking studies between DC-SIGN monomer and OSR1. It is possible to see residues **L** and **G** in the binding pocket while the rest are solvent exposed. B) RMSD of the MD simulation of the DC-SIGN monomer (black) and OSR1 pentasaccharide (red) calculated having the protein as reference. The protein is stable while it is possible to observe how after a few nanoseconds the ligand leaves the binding pocket.

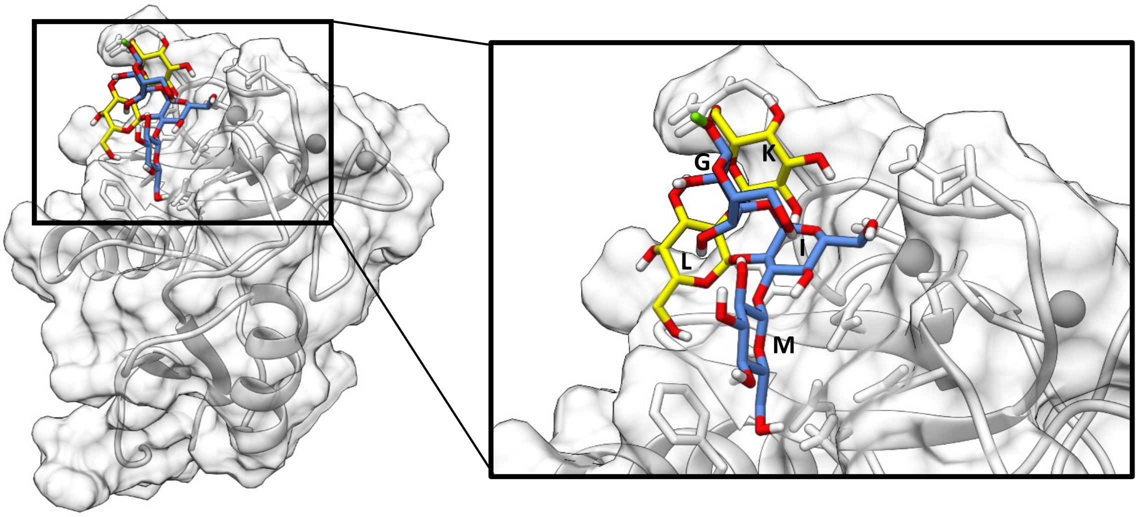

Figure S4. Representative pose of the most populated cluster (cluster B) from the docking studies between DC-SIGN monomer and OSR1 outer core pentasaccharide. Related to Figure 4. Residues **K**, **I**, and **M** are in the binding pocket while **L** and **G** are solvent exposed.

A

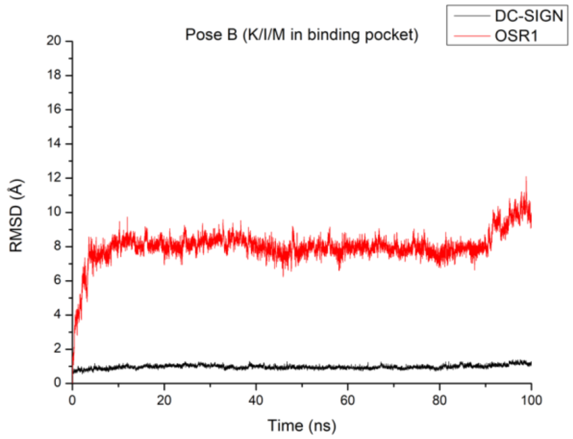

B

Acceptor	DonorH	AvgDist	%
2LA_K@O6	ASN_362@H	2,8570	68,70
SER_360@OG	ZGA_I@H6O	2,7967	44,96
2LA_K@O4	ARG_345@H	2,9110	34,89
0GB_M@O3	ASN_362@HD21	2,8773	20,21
ASN_344@OD1	2LA_K@H6O	2,8077	20,15
ASN_311@OD1	0GB_M@H6O	2,7374	10,51

C

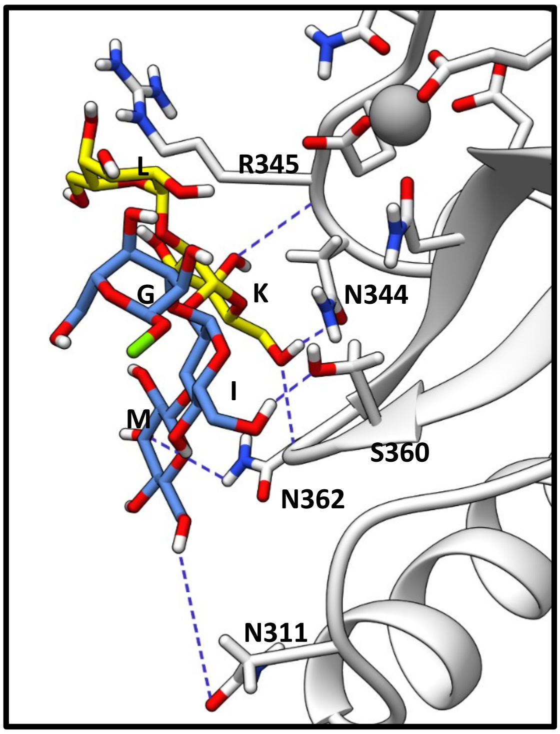

Figure S5. **Cluster B MD simulation studies. Related to Figure 4.** A) RMSD of the DC-SIGN monomer (black) and OSR1 pentasaccharide (red) calculated having the protein as reference. The protein is stable while the ligand after few nanoseconds shifts from the binding pocket to interact with a polar region. B) Protein-ligand H-bonds described as acceptor, donor, distance and stability (in percentage) during the 100 ns MD coloured according to the ligand residue involved in the interaction. C) Representative pose of the interaction between DC-SIGN monomer protein and OSR1 pentasaccharide. The ligand is coloured according to the SNFG nomenclature. Ca^{2+} ion is coloured in grey. In green the linking point between residue **G** and the rest of the LOS.

A

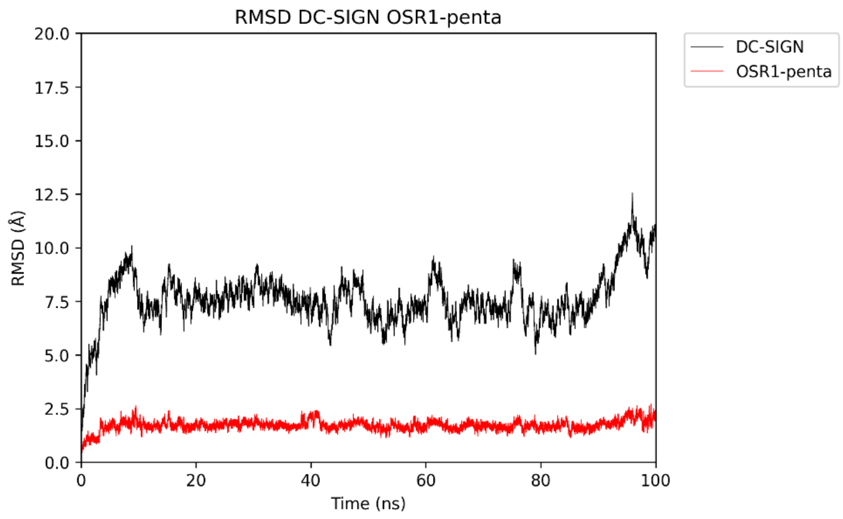

B

#Acceptor	DonorH	AvgDist	%
GLU_347@OE2	Glc_G@H6O	2,6793	91
ASN_362@OD1	Glc_M@H2O	2,7241	73
GLU_354@OE2	Gal_L@H3O	2,6495	56
ASN_344@OD1	Gal_K@H6O	2,7616	53
Gal_L@O3	ASN_365@HD21	2,8834	48
Gal_K@O6	ASN_362@H	2,8893	43
Glc_I@O6	SER_360@HG	2,7751	21
Glc_M@O3	ASN_362@HD21	2,8802	20
Glc_G@O5	ASN_344@HD22	2,8800	15
GLU_347@OE2	Gal_L@H4O	2,7123	14
ASN_311@OD1	Glc_M@H6O	2,7380	13
SER_360@OG	Glc_I@H6O	2,8128	12

Figure S6 Analysis of the interaction and stability of OSR1 recognition by two DC-SIGN units. Related to Figure 4 and 5. A) DC-SIGN (black) and OSR1-pentasaccharide (red) RMSD. The ligand RMSD was calculated in reference to the protein. B) Protein-ligand H-bonds described as acceptor, donor, distance and stability (in percentage) during the 100 ns MD.

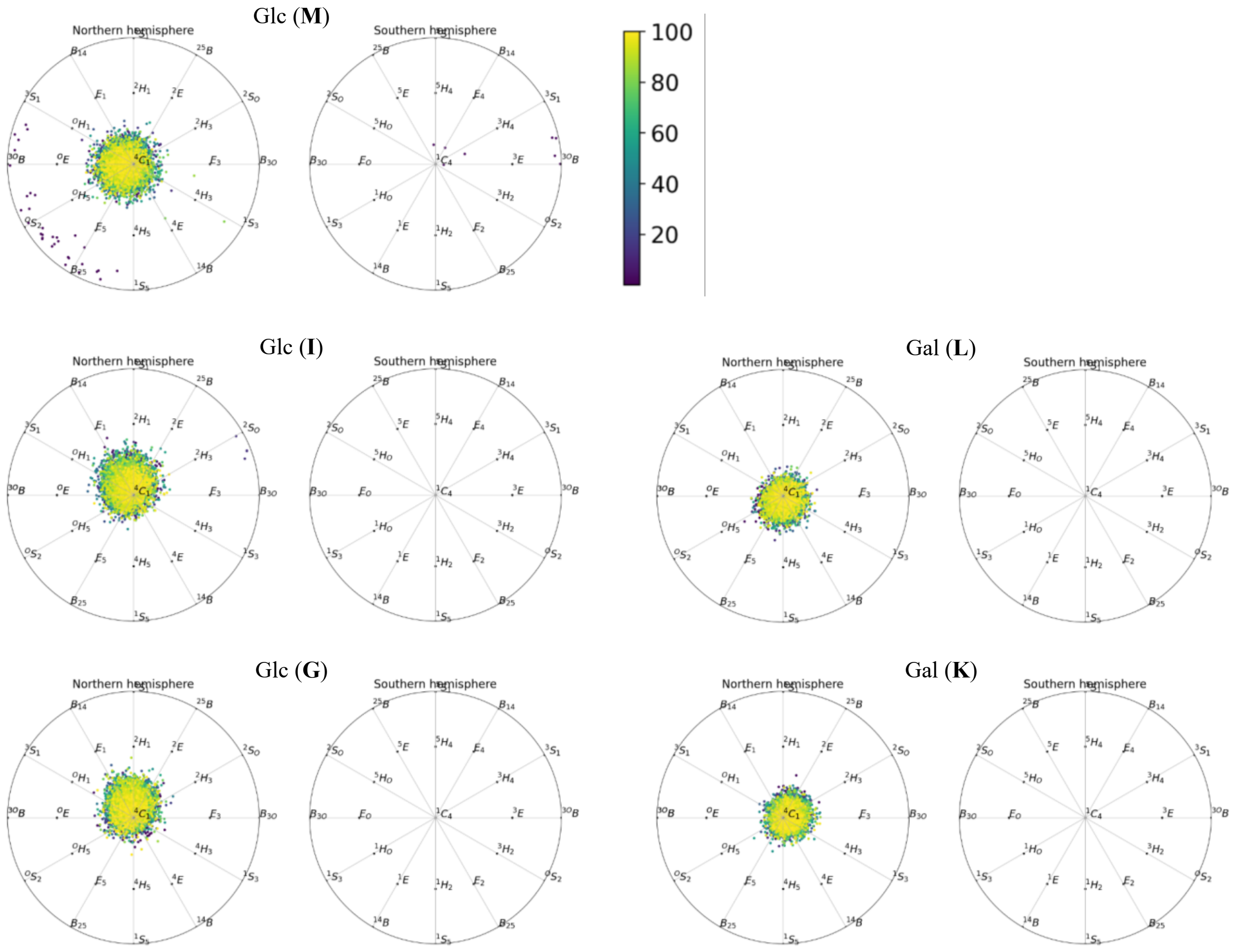

Figure S7 Conformational analysis of the pentasaccharide composing sugars along the MD simulation. Related to Figure 4. Northern and Southern hemisphere of the Stoddart diagram representing the distribution of conformations during the 100 ns MD simulation for the 5 residues of the outer core pentasaccharide. In all the cases the $^4C_1$ conformation is maintained. The script used to obtain the Stoddart diagrams was kingly provided by Prof. Carme Rovira group.

β-Glc-(1-3)-α-Glc
(M-I)

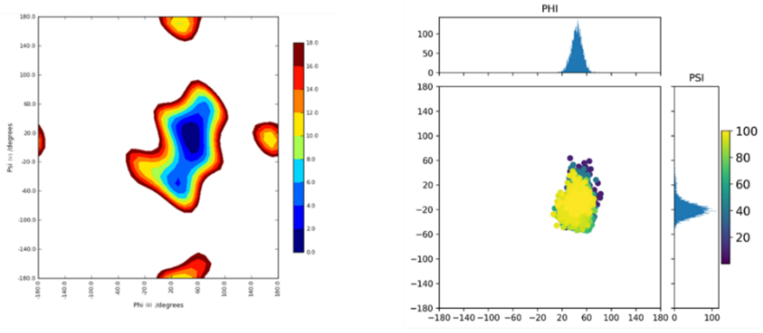

α -Glc-(1-3)-α-Glc
(I-G)

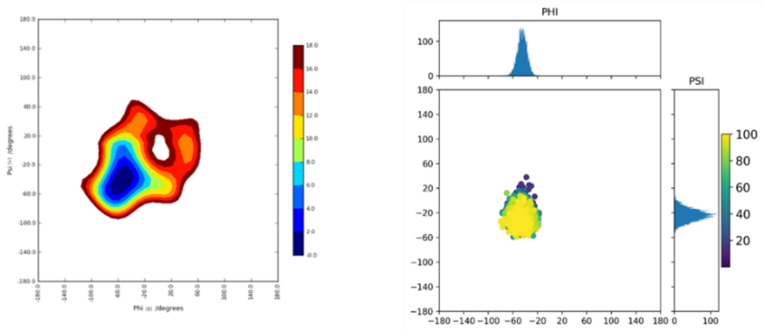

α -Gal-(1-2)-α-Gal
(L-K)

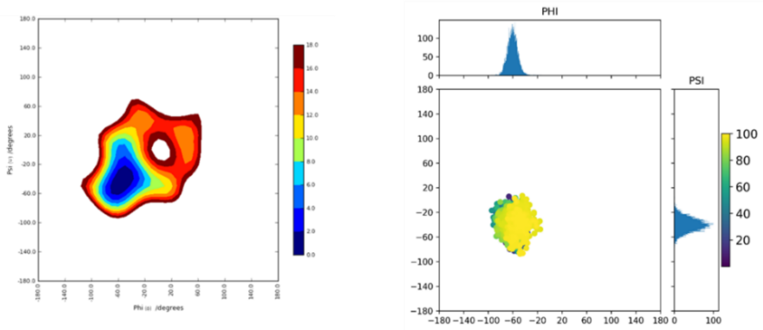

α -Gal-(1-2)-α-Glc
(K-I)

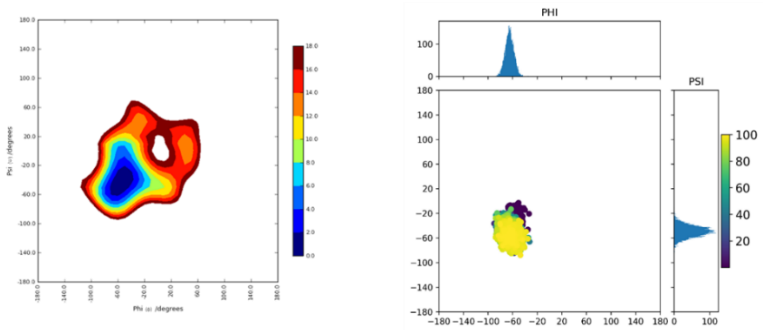

Figure S8. Conformational study of the pentasaccharide glycosidic linkages during the MD simulation. Related to Figure 4. Adiabatic energy maps, obtained by molecular mechanics calculations, illustrating the energetic minima of the glycosidic torsion angles ϕ/ψ of the outer core LOS R1 constituent disaccharides. Outer core pentasaccharide dihedral angles analysis in the bound state represented as scatter plots of the ϕ torsion against ψ, during the MD simulation with the relative histograms to represent the most populated energies.
